# Training Dementia Care Partners in Rural China: A Mixed-Methods Randomized Controlled Feasibility Trial

**DOI:** 10.1093/geroni/igaf122.4218

**Published:** 2025-12-31

**Authors:** Dexia Kong

**Affiliations:** The Chinese University of Hong Kong, Hong Kong, Hong Kong, China (People’s Republic)

## Abstract

China has the world’s largest dementia population. Despite a higher prevalence of dementia in rural areas, few dementia family caregiver training intervention studies have been conducted in rural China. To address this gap, we conducted a mixed-methods RCT feasibility trial. In Phase 1, we conducted in-depth qualitative interviews with 25 local family caregivers regarding their caregiving experiences and needs, alongside focus group discussions with 30 local healthcare providers exploring their caregiving experiences, to inform the cultural adaptation of the World Health Organization’s iSupport program to fit the rural Chinese context. In Phase 2, we implemented a two-arm randomized controlled feasibility trial (n = 64) to evaluate the preliminary effectiveness and feasibility of the adapted program delivered via face-to-face group sessions (Arm 1) versus a wait-list control group (Arm 2). Treatment outcomes, including dementia knowledge and caregiving self-efficacy, and caregiver burden, were measured at baseline (T1), 2 months after baseline (T2), and 4 months after baseline (T3), using validated instruments. Feasibility indicators included retention rate and treatment usability. A subset of participants (n = 25) participated in post-treatment qualitative interviews to understand their program experiences, perceived impacts, and barriers. Intention-to-treat analysis indicates promising preliminary effects on knowledge boost and burden reduction. The training program had high retention rate (81%) and usability scores (mean = 2.8, range: 0-4). Results from both phases suggest the program’s potential scalability. The findings on the initial effectiveness of the culturally-adapted caregiver training program can inform key ingredients for a future full-scale pragmatic trial in low-resource settings.

